# Diagnostic and prognostic accuracy of clinical and laboratory parameters in community-acquired pneumonia

**DOI:** 10.1186/1471-2334-7-10

**Published:** 2007-03-02

**Authors:** Beat Müller, Stephan Harbarth, Daiana Stolz, Roland Bingisser, Christian Mueller, Jörg Leuppi, Charly Nusbaumer, Michael Tamm, Mirjam Christ-Crain

**Affiliations:** 1 From the Departments of Internal Medicine, University Hospital, Petersgraben 4, CH-4031, Basel, Switzerland; 2 Division of Hospital Epidemiology, University Hospital, CH-1211, Geneva, Switzerland; 3 Department of Pneumology, University Hospital, Petersgraben 4, CH-4031, Basel, Switzerland; 4 Emergency Department, University Hospital, Petersgraben 4, CH-4031, Basel, Switzerland; 5 Department of Cinical Chemistry, University Hospital, Petersgraben 4, CH-4031, Basel, Switzerland

## Abstract

**Background:**

Community-acquired pneumonia (CAP) is the most frequent infection-related cause of death. The reference standard to diagnose CAP is a new infiltrate on chest radiograph in the presence of recently acquired respiratory signs and symptoms. This study aims to evaluate the diagnostic and prognostic accuracy of clinical signs and symptoms and laboratory biomarkers for CAP.

**Methods:**

545 patients with suspected lower respiratory tract infection, admitted to the emergency department of a university hospital were included in a pre-planned post-hoc analysis of two controlled intervention trials. Baseline assessment included history, clinical examination, radiography and measurements of procalcitonin (PCT), highly sensitive C-reactive protein (hsCRP) and leukocyte count.

**Results:**

Of the 545 patients, 373 had CAP, 132 other respiratory tract infections, and 40 other final diagnoses. The AUC of a clinical model including standard clinical signs and symptoms (i.e. fever, cough, sputum production, abnormal chest auscultation and dyspnea) to diagnose CAP was 0.79 [95% CI, 0.75–0.83]. This AUC was significantly improved by including PCT and hsCRP (0.92 [0.89–0.94]; p < 0.001). PCT had a higher diagnostic accuracy (AUC, 0.88 [0.84–0.93]) in differentiating CAP from other diagnoses, as compared to hsCRP (AUC, 0.76 [0.69–0.83]; p < 0.001) and total leukocyte count (AUC, 0.69 [0.62–0.77]; p < 0.001). To predict bacteremia, PCT had a higher AUC (0.85 [0.80–0.91]) as compared to hsCRP (p = 0.01), leukocyte count (p = 0.002) and elevated body temperature (p < 0.001). PCT, in contrast to hsCRP and leukocyte count, increased with increasing severity of CAP, as assessed by the pneumonia severity index (p < 0.001).

**Conclusion:**

PCT, and to a lesser degree hsCRP, improve the accuracy of currently recommended approaches for the diagnosis of CAP, thereby complementing clinical signs and symptoms. PCT is useful in the severity assessment of CAP.

## Background

Community-acquired pneumonia (CAP) is the major infection-related cause of death in developed countries [1,2]. The reference standard to diagnose CAP is a new infiltrate on chest radiograph in the presence of recently acquired respiratory signs and symptoms [2-4]. These include cough, increased sputum production, dyspnea, fever and abnormal auscultatory findings [5]. Unfortunately, clinical findings do not reliably predict radiologically confirmed pneumonia [6]. Especially elderly people often present with atypical symptoms and without fever [7]. Physicians, especially in primary care, may not perform radiography and rely on the patient's history and physical examination [8].

The differential diagnosis of CAP includes several non-infectious causes, including pulmonary embolism, malignancy and congestive heart failure, among others [9]. The presence of a non-infectious differential diagnosis is usually suspected only after failure of antibiotic therapy, with the ensuing risks related to untreated, potentially life-threatening non-bacterial disease [10]. Conversely, a delay of antibiotic treatment of more than 4 hours after hospital admission is associated with increased mortality [11]. Hence, both a rapid diagnosis of CAP and an accurate differentiation from viral respiratory illnesses and non-infectious causes has important therapeutic and prognostic implications [12].

We recently reported the results of 2 intervention trials that assessed the value of procalcitonin (PCT) in guiding antibiotic treatment decisions in consecutive patients with suspected lower respiratory tract infections [13,14]. In the present pre-planned, post-hoc analysis of the 2 datasets containing detailed data from 545 patients, we evaluated three clinically relevant questions. To mirror an approach often done in primary care, we first evaluated the diagnostic accuracy of different parameters for diagnosing CAP solely based on history, clinical examination and laboratory parameters without radiography. To mirror an approach in an emergency department, we secondly compared different parameters to differentiate bacterial CAP from other differential diagnoses in patients with suspected CAP based on recently acquired respiratory signs and an infiltrate in chest radiograph. Third, we estimated the accuracy of different parameters to predict bacteremia and the severity of CAP.

## Methods

### Setting and Study Population

Data from two randomized prospective studies with a total of 545 patients with suspected lower respiratory tract infections, who presented to the emergency department of a 950-bed tertiary care center in Basel (Switzerland), were combined in a preplanned post-hoc analysis. The design of the two studies was similar. A complete description has been reported elsewhere [13,14]. In brief, consecutive patients with clinically suspected lower respiratory tract infections [13] and radiologically confirmed CAP [14], respectively, admitted from December 2002 until April 2003 (n = 243) and from November 2003 through February 2005 (n = 302) to the University Hospital were analyzed. The primary endpoint of the two studies was to evaluate the prescription and duration of antibiotic use in patients randomly assigned to PCT-guidance (275 patients) as compared to standard recommended guidelines (270 patients). Patients had to be > 18 years with a suspected lower respiratory tract infection (i.e. CAP, acute exacerbations of chronic obstructive pulmonary disease [AECOPD], acute bronchitis, asthma exacerbation) as principal diagnosis on admission. CAP was defined by the presence of one or several of the following recently acquired respiratory signs and symptoms: cough, sputum production, dyspnea, core body temperature ≥ 38.0°C, auscultatory findings of abnormal breath sounds and rales, leukocyte count > 10 or < 4 × 10^9 ^cells L^-1 ^and an infiltrate on chest radiograph [2]. AECOPD was defined as a FEV1/FVC ratio below 70% and the severity of AECOPD was defined as proposed[15] Acute bronchitis was defined as acute onset cough of 2 to 14 days with or without sputum production in the absence of an underlying lung disease or focal chest signs and infiltrates on chest radiography, respectively [16]. Asthma was defined as episodic symptoms of airflow obstruction, at least partially reversible as assessed by lung function tests [17]. Excluded were patients with cystic fibrosis or active pulmonary tuberculosis; hospital-acquired pneumonia and severely immunocompromised patients (e.g. AIDS, febrile neutropenia after chemotherapy).

All patients were examined on admission to the emergency department by a resident supervised by a board-certified specialist in internal medicine. Baseline assessment included clinical data and vital signs, comorbid conditions, and routine blood tests. Chest radiographs were screened by the physician in charge. A senior radiologist, unaware of clinical and laboratory findings, reviewed all chest radiographs. The Pneumonia Severity Index (PSI) and the CURB65 score were calculated as described [18,19].

The patients' functional status was assessed using a visual analogue scale, ranging from 0 (feeling extremely ill) to 100 (feeling completely healthy), and by a quality of life (QoL) questionnaire for patients with respiratory illnesses [12].

Both studies were approved by the local Ethics Committee (Ethikkommission beider Basel, "EKBB") and all participants gave written informed consent to participate in the study. After publication of the novel rules [20], the second trial was registered in the Current Controlled Trials Database [ISRCTN04176397] [21].

### Definition of the presence and absence of a bacterial cause in suspected CAP

A microorganism was defined as causing agent, if detected in respiratory specimens (sputum or bronchoalveolar lavage fluid [BAL]), blood, or both, excluding normal skin or mucosal flora. Only relevant microorganisms cultured from representative sputum specimens according to Murray's criteria were considered, i.e., > 25 leukocytes and < 10 epithelial cells per high power field. Identical microorganisms cultured in a patient from both sputum and bronchoalveolar lavage fluid were considered only once. We searched for *Legionella pneumophila *antigen in urine (Legionella now Binax, Portland, ME, USA), by culture or by polymerase chain reaction (PCR) from bronchoalveolar lavage fluid. *Mycoplasma pneumoniae *was detected by culture or PCR in BAL fluid. *Chlamydia pneumoniae *was identified by PCR in BAL fluid. As used to define bacterial CAP in a previous study [22] clinically relevant bacterial CAP was considered to be absent if an alternative cause for pulmonary infiltrate was established without bacterial growth in culture results or if the patient completely recovered from fever, infiltrates, and leukocytosis without appropriate antimicrobial therapy.

### Measurement of Biomarkers

PCT was measured within one hour after blood sampling using 20 to 50 μL of plasma or serum by a time-resolved amplified cryptate emission (TRACE) technology assay (Kryptor^® ^PCT, Brahms AG, Hennigsdorf, Germany), as described [13]. The assay has a functional assay sensitivity of 0.06 μg/L, which is about four-fold above mean normal levels [23]. Highly-sensitive C-reactive protein (hsCRP) was measured in heparin plasma on a Hitachi Instrument 917 (Roche Diagnostics, Rotkreuz, Switzerland; using reagents provided by Wako Chemicals GmbH, Neuss, Germany). Levels that were non-detectable were assigned a value equal to the lower limit of detection for the assay.

In 74 and 80 patients, PCT and CRP, respectively, were measured not only on admission, but also after 6 to 24 hours. This follow-up measurement was foreseen in the study protocols for all cases with initial uncertainty. In these patients, the peak values were used for the analyses.

### Statistical Analysis

Discrete variables are expressed as counts (percentage) and continuous variables as means ± standard deviation (SD), unless stated otherwise. Frequency comparison was done by chi-square test. Two-group comparison of normally distributed data was performed by Student's t-test. For multigroup comparisons, one-way analysis of variance (ANOVA) with least square difference for posthoc comparison was applied. For data not normally distributed, the Mann-Whitney-U test was used if only two groups were compared and the Kruskal-Wallis one-way analysis of variance was used if more than two groups were being compared. Correlation analyses were performed by using Spearman rank correlation. Standard definitions of sensitivity, specificity, and likelihood ratio (LR) were used [24,25]. We constructed receiver operating characteristic (ROC) curves and determined the areas under the receiver operating characteristic curve (AUC), as previously described [26-28]. All statistical tests were 2-tailed. P < 0.05 was considered significant. Statistical packages used were MedCalc for Windows, (version 7.2.1.0., Mariakerke, Belgium) and STATA, version 8.0 (Stata, College Station, USA).

## Results

### Baseline characteristics of the patients

Baseline characteristics of the 545 patients on admission are shown in Table 1.

**Table 1:** Baseline Characteristics of the 545 Patients*

**Characteristic**	
Age – years	67.1 ± 18.0
Male sex – no. (%)	314 (62.6)
**Smoking status**	
- Current smoker – no. (%)	135 (24.8)
- Packyears history in smokers	40.0 ± 24.9
**Antibiotic pretreatment (%)**	110 (20.2)
**Underlying disease **– no. (%)	
- Coronary artery disease	156 (28.6)
- Hypertensive heart disease	95 (17.4)
- Congestive heart failure	34 (6.2)
- Peripheral vascular disease	39 (7.2)
- Cerebrovascular disease	25 (4.6)
- Renal dysfunction	121 (22.2)
- Liver disease	43 (7.9)
- Diabetes mellitus	93 (17.1)
- Neoplastic disease	65 (11.9)
**Symptoms **– no. (%)	
- Cough	497 (91.2)
- Sputum	390 (71.6)
- Dyspnea	392 (71.9)
**Signs– **no. (%)	
- Rales	394 (72.3)
**Final diagnoses **– no. (%)	
- CAP	373 (68.4)
- Other respiratory tract infections	132 (24.2)
- Asthma	13 (2.4)
- Acute bronchitis	59 (10.8)
- Acute exacerbation of COPD	60 (11.0)
- Others	40 (7.3)
- With infiltrate on chest radiography	23 (4.2)
- Without infiltrate on chest radiography	17 (3.1)
**PSI in patients with CAP **– points	96.4 ± 36.0
**PSI class **– no. (%)	
- I, II and III	162 (43.4)
- IV	150 (40.2)
- V	61 (16.4)
**Laboratory parameters**	
hsCRP (mg/L) (mean; median (range))	127.9; 103.4 (0.5–512)
PCT (μg/L) (mean; median (range))	3.1; 0.32 (0.02–234.7)
Leukocyte count (×10^9^/L)	12.9 ± 6.7

*Plus-minus values are means ± SD. Because of rounding, percentages may not sum to 100. COPD denotes chronic obstructive pulmonary disease, PSI pneumonia severity index, CAP community-acquired pneumonia, hsCRP highly-sensitive C-reactive protein, PCT procalcitonin.

Temperature > 37.9°C was present in 290 (53.2%) patients. The typical triad of cough, fever and dyspnea, as reported by the patient, was present in 230 (42.2%) of cases. Abnormal chest auscultation was present in 469 (86.1%) patients, rales in 394 (72.3%) patients.

396 (72.7%) patients had an infiltrate on chest radiography and were on admission classified as having CAP. In 373 (68.4%) CAP was confirmed as final diagnosis at follow-up. Conversely, 23 (4.2%) patients with infiltrate on chest radiography had a final diagnosis other than lower respiratory tract infection. This included 20 (3.7%) patients with a non-infectious final diagnosis, i.e. congestive heart failure (4), pulmonary embolism (4), cryptogenic organizing pneumonia (2), malignancy (5), unknown interstitial pneumopathy (2), pleural effusion of unknown etiology (1), polyserositis (1), Wegener granulomatosis (1). In addition, 3 patients with infiltrate on chest radiography had other infectious diagnoses, namely, urosepsis (1), endocarditis (1), deep surgical site infection of the sternum (1). 17 (3.1%) patients without infiltrate on chest radiography had a diagnose other than lower respiratory tract infections.

### Microbiology

Overall, in the 373 patients with the final diagnosis of CAP, 11.3% of blood cultures and 21.5% of cultures from respiratory secretions were positive. Overall the responsible pathogen in patients with CAP could be cultured in 98 (26.3%) of patients. The causative microorganisms are listed in Table 2. The most frequently isolated microorganism was *Streptococcus pneumoniae*.

**Table 2:** Identified microorganisms in patients with CAP (n = 373)

	**Respiratory secretions**	**Blood cultures**
**Overall positive **– no. (%)	80 (21.5)	42 (11.3)
		
**Gram-positive organisms**	**39**	**36**
*Streptococcus pneumoniae*	32	33
*Streptococcus milleri*	2	0
*Staphylococcus aureus*	4	3
*Enterococcus *species	1	0
		
**Gram-negative organisms**	**34**	**6**
*Moraxella catarrhalis*	3	0
*Haemophilus influenzae*	9	0
*Pseudomonas *species	13	2
*Klebsiella *species	7	2
*Neisseria meningitidis*	0	2
Morganella morganii	1	0
Enterobacteriaceae	1	0
		
**Atypical pathogens**	**7**	**0**
*Mycoplasma pneumoniae*	2	0
*Legionella pneumophila *‡	5	0

‡ *Legionella pneumophilia *was detected by urinary antigen in 4 patients.

### Diagnostic accuracy for discriminating CAP from other lower respiratory tract infection without radiography

To mirror an approach occasionally done in primary care, we evaluated the diagnostic accuracy for diagnosing CAP (n = 373) solely based on history, clinical examination and laboratory parameters without ascertainment by radiography.

**Figure 1:**
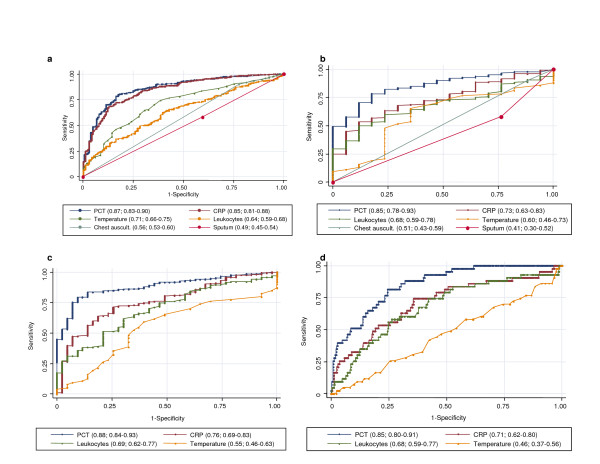
**Receiver operating characteristics curves (ROC) of different parameters for the diagnosis of pneumonia**. **a **diagnostic accuracy to predict CAP without chest radiography: Primary care approach. **b **diagnostic accuracy to predict radiographically suspected CAP (control group (n = 20) includes other non-infectious diagnoses initially diagnosed as CAP): Emergency department approach. **c **diagnostic accuracy to predict radiographically suspected CAP (control group (n = 44) includes other non-infectious diagnoses initially diagnosed as CAP (n = 20) plus patients without a clinically relevant bacterial etiology of CAP (n = 24). **d **diagnostic accuracy to predict bacteremic CAP. Values show areas under the ROC curve with 95% confidence intervals. Chest auscult. denotes abnormal chest auscultation; CRP C-reactive Protein; PCT procalcitonin.

As shown in Figure 1a, PCT and hsCRP yielded the highest discriminative value to diagnose CAP. In this setting, the difference between hsCRP and PCT was not significant (p = 0.36). In comparison, commonly used clinical signs such as fever (temperature > 37.9°C), leukocyte count, an abnormal chest auscultation, sputum production, dyspnea and cough had all lower discriminative values (p for all comparisons of PCT and hsCRP, respectively, with other parameters < 0.001).

A clinical model including fever, cough, sputum production, abnormal chest auscultation and dyspnea [5] had an AUC of 0.79 (95% CI, 0.75–0.83). The inclusion of hsCRP in this clinical model increased this AUC value to 0.90 (95% CI, 0.87–0.93; p < 0.001). The inclusion of PCT increased the AUC of the clinical model to 0.88 (0.85–0.91; p < 0.001). The clinical model with hsCRP was not significantly different as compared to that with PCT (p = 0.18). A clinical model including both hsCRP and PCT had an AUC of 0.92 (95% CI, 0.89–0.94), which was significantly better as compared to a model using PCT or hsCRP alone (p < 0.001 for both comparisons), respectively.

The multilevel likelihood ratios for hsCRP and PCT levels in diagnosing CAP are shown in Table 3.

**Table 3:** Multilevel likelihood ratios for hsCRP and PCT to diagnose CAP without chest radiography

	**N (%)**	**Sensitivity**	**Specificity**	**LR +**	**LR -**
**PCT (μg/L)**					
> 0.1	406 (75)	0.90	0.59	2.22	0.16
> 0.25	300 (55)	0.74	0.85	4.87	0.31
> 0.5	225 (41)	0.57	0.93	8.21	0.46
> 1.0	167 (31)	0.43	0.96	10.57	0.59
**hsCRP (mg/L)**	**N (%)**	**Sensitivity**	**Specificity**	**LR +**	**LR -**
> 40	413 (76)	0.89	0.52	1.86	0.22
> 50	384 (70)	0.87	0.65	2.44	0.21
> 100	281 (52)	0.69	0.86	4.94	0.36
> 200	141 (26)	0.36	0.96	8.83	0.67

All results were similar after age stratification for patients > 75 years.

### Diagnostic accuracy for radiographically defined CAP

Herein, we evaluated the diagnostic accuracy for differentiating radiographically and clinically diagnosed CAP from other differential diagnoses of CAP.

First, to separate the 20 (3.7%) patients with an infiltrate on chest radiography and a non-infectious final diagnosis from the 373 confirmed CAP patients, the diagnostic accuracy of PCT was higher as compared to hsCRP (p = 0.04), leukocyte count (p = 0.01), body temperature (p = 0.001), chest auscultation (p < 0.001) and sputum production (p < 0.001) (Figure 1b).

Second, 24 (6.4%) of the 373 patients with the final diagnosis of CAP fulfilled the criterion of full recovery from fever, infiltrates, and leukocytosis without any antimicrobial therapy. As suggested as definition for bacterial CAP in a previous study [22] these 24 patients were thus classified as not having pneumonia of clinically relevant bacterial origin. After adding these 24 patients to the 20 patients with a proven non-infectious origin of chest infiltrates, the diagnostic accuracy for PCT was also higher as compared to hsCRP (p < 0.001), leukocyte count (p < 0.001) and body temperature (p < 0.001, Figure 1c).

The multilevel likelihood ratios for hsCRP and PCT levels in this setting are shown in Table 4.

**Table 4:** Multilevel likelihood ratios for hsCRP and PCT to diagnose CAP in patients with an infiltrate on chest radiography

	**N (%)**	**Sensitivity**	**Specificity**	**LR +**	**LR -**
**PCT (μg/L)**					
> 0.1	349 (89)	0.90	0.39	1.48	0.25
> 0.25	280 (71)	0.74	0.74	2.83	0.35
> 0.5	217 (55)	0.57	0.83	3.30	0.52
> 1.0	163 (41)	0.43	0.87	3.31	0.65
**hsCRP (mg/L)**	**N (%)**	**Sensitivity**	**Specificity**	**LR +**	**LR -**
> 40	349 (89)	0.89	0.17	1.07	0.65
> 50	339 (86)	0.87	0.26	1.17	0.52
> 100	265 (67)	0.69	0.61	1.76	0.51
> 200	136 (34)	0.36	0.91	4.14	0.70

### Diagnostic accuracy to predict bacteremia

Of the 373 patients with the final diagnosis of CAP, 42 (11.3%) had positive blood cultures. Thus, a positive blood culture had a sensitivity of 11.3 percent to predict CAP. To predict bacteremia in patients with CAP, PCT had a higher AUC as compared to hsCRP (p = 0.01), leukocyte count (p = 0.002) and elevated body temperature (p < 0.001) (Figure 1d). The multilevel likelihood ratios for hsCRP and PCT levels in diagnosing bacteremia in patients with CAP in this setting are shown in Table 5.

**Table 5:** Multilevel likelihood ratios for hsCRP and PCT to diagnose bacteremia in patients with CAP

	**N (%)**	**Sensitivity**	**Specificity**	**LR +**	**LR -**
**PCT (μg/L)**					
> 0.1	336 (90)	1.0	0.11	1.12	< 0.01
> 0.25	274 (74)	0.98	0.29	1.38	0.08
> 0.5	213 (57)	0.93	0.48	1.78	0.14
> 1.0	160 (43)	0.86	0.63	2.32	0.22
**hsCRP (mg/L)**	**N (%)**	**Sensitivity**	**Specificity**	**LR +**	**LR -**
> 40	331 (89)	0.91	0.12	1.03	0.79
> 50	323 (87)	0.91	0.14	1.06	0.65
> 100	257 (69)	0.86	0.33	1.30	0.41
> 200	134 (36)	0.64	0.68	1.98	0.54

### Accuracy to predict severity of CAP

**Figure 2:**
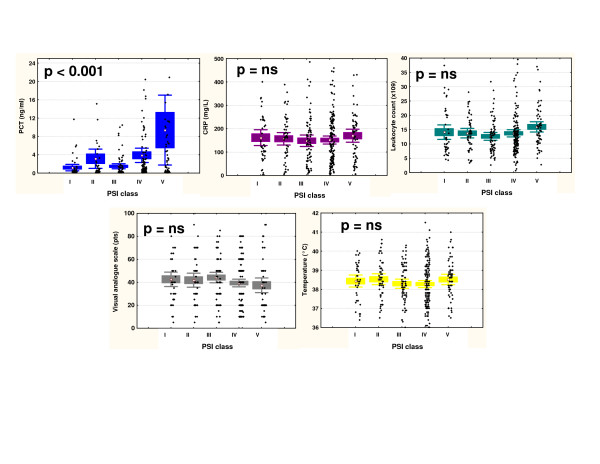
**PCT as compared to hsCRP and leukocyte count in different severities of CAP**. PCT denotes procalcitonin, CRP highly-sensitive C-reactive protein, PSI pneumonia severity index. Diamonds denote means, squares SEM and whiskers 1.96 SEM of the combined data. The scatterplots represent all values.

PCT levels increased with increasing severity of CAP, classified according to the PSI score (p < 0.001). This increase was more pronounced as compared to total leukocyte count (p = 0.08), C-reactive protein (p = 0.90), body temperature (p = 0.42), and the visual analogue scale (p = 0.21) (Figure 2). Results for the CURB65 score were similar (data not shown). PCT levels in patients with mild CAP (defined as PSI class I to III) were significantly lower as compared to patients with severe CAP (defined as PSI class IV and V; p < 0.001). This difference was not significant for C-reactive protein (p = 0.96), total leukocyte count (p = 0.25), body temperature (p = 0.48) and the visual analogue scale (p = 0.06).

## Discussion

Our data show that clinical signs and symptoms routinely used and recommended to screen and to establish the diagnosis of CAP are of limited value. Both, PCT and hsCRP improve the diagnostic value of the clinical assessment. On admission, PCT has the highest diagnostic accuracy in differentiating radiologically confirmed CAP from other differential diagnoses, to predict the later finding of bacteremia and to assess the severity of CAP.

According to most guidelines, an infiltrate in chest radiograph is mandatory for the diagnosis of CAP. However, in the setting of primary care, radiography may often not be performed [8]. Instead, based on the clinical suspicion and in view of the severe consequences of delayed antibiotic therapy, physicians have a low threshold to initiate antibiotic therapy in presumed CAP [29,30]. Obviously, the timely clinical evaluation of patients with symptoms suggestive of CAP is important to estimate the pretest probability for the disease, to assess the severity of illness, and to start appropriate antimicrobial therapy. However, as confirmed by our data, most routinely used clinical parameters, alone or in combination, have poor diagnostic accuracies to predict CAP. Similar clinical signs and symptoms are caused by acute bronchitis, AECOPD, asthma exacerbations and non-infectious diagnoses such as congestive heart failure or atelectasis. In addition, the interpretation of the clinical assessment lacks standardization and validation and is, therefore, prone to interobserver variability [31,32]. We are aware that the lack of diagnostic accuracy of history and clinical examination has been reported [5,31]. However, despite their known limitations, clinical signs are daily used for decision making in clinical routine. Herein, we propose the use of biomarkers to complement and improve clinical assessment.

As shown by our data, together with a careful clinical assessment and radiology, biomarkers like PCT and hsCRP can significantly increase diagnostic accuracy for the diagnosis of CAP. One might argue that the diagnosis of bronchitis, CAP and AECOPD should be clinically evident as should the indication for antibiotic therapy. However, despite the presence of excellent and recent guidelines, the implementation into clinical routine namely for these "evident diseases" is insufficient. Thus in our opinion, as a biomarker PCT becomes especially valuable as a powerful tool to better complement and implement these guidelines, as shown in our intervention studies.

In the setting of an emergency department of a hospital, most patients presenting with symptoms of respiratory tract infection will receive a chest radiograph. In the presence of a new infiltrate, a bacterial etiology is usually assumed. However, bacterial causes must be differentiated from other, non-infectious or viral etiologies, which can be challenging. We demonstrate that the routinely used clinical parameters in radiographically defined CAP miss the diagnostic accuracy to differentiate between bacterial and non-bacterial CAP. PCT, better than hsCRP, improves diagnostic accuracy to distinguish bacterial CAP from non-infectious or non-bacterial causes, respectively. Thus, if a patient shows an infiltrate on chest radiograph in the presence of acute respiratory symptoms and very low PCT levels (< 0.1 μg/L), clinicians should actively seek for an alternative diagnosis to bacterial pneumonia.

Positive blood cultures in CAP patients correlate with adverse outcome and, thus, a rapid initiation of antimicrobial treatment is pivotal [33-35]. However, results from microbiological cultures of body fluids are only available after 24 to 48 hours, which can be problematic for clinical practice. A PCT cut-off of 0.25 μg/L had a 98% sensitivity to detect bacteremia. PCT may thus provide valuable and faster information about severity of disease long before blood culture results become available. Accordingly, PCT better mirrored the severity of CAP classified by the PSI, as compared to hsCRP levels, leukocyte counts or the visual analogue scale, which were unable to distinguish mild from more severe pneumonia.

Several limitations of our study merit consideration. First, results of two studies were combined introducing the possibility of a selection bias, although in both studies the primary endpoint was similar. Second, since antibiotics were withheld based on PCT levels, this may have introduced a bias to the favor of PCT. Conversely, cure of CAP under antibiotic therapy may falsely have been considered as proof of bacterial etiology in a considerable proportion of patients who indeed had a non-bacterial etiology. Third, our results may not apply for immunosuppressed patients and other clinical settings or sites of infection, especially localized or fungal pulmonary infections such as empyema and aspergillosis. However, these are unlike conditions in outpatients presenting with lower respiratory tract infections. The diagnostic accuracy of PCT in patients with immunosuppression or hospital-acquired pneumonia has to be evaluated in future studies. These patients were excluded for safety reasons. Forth, interobserver variation in the clinical evaluation of patients with CAP has not been examined. Other studies have revealed considerable interobserver variability in the recording and evaluation of symptoms. Fifth, we only assessed total leukocyte count and not band forms. However, in recent studies, the superiority of hsCRP and PCT as compared to leukocyte count and band counts has been shown [25,36,37]. Finally, the rate of microbiologically documented CAP in our study population was rather low, limiting information about the diagnostic accuracy of PCT for the etiological diagnoses. We did not routinely perform serology or PCR or culture in blood and respiratory secretions for *Mycoplasma pneumoniae *and *Chlamydia pneumoniae*. Moreover, search for *Streptococcus pneumoniae *antigen in urine was not routinely done. However, using representative respiratory secretions and blood cultures the rate of documented bacterial CAPs in our study was very similar to the one in different recent studies [38] or [39].

Strengths of our study are first that the study population included a relatively diverse group of patients with lower respiratory tract infections. Second, we did not include clinically unrealistic control patients without suspected infection, but only patients with a high pretest probability of CAP, covering the spectrum that is likely to be encountered in the future use of these tests [40]. Our study is thus based on a real-life patient sample to closely resemble clinical practice in a emergency room setting. As compared to primary care setting, our study cohort might have a higher pretest probability for pneumonia which might bias our question about the diagnostic accuracy of PCT for diagnosing CAP solely based on history, clinical examination and laboratory parameters without radiography, an approach occasionally done in primary care.

Finally, the rational in our trials was the concept that diagnosis is not the principle outcome measure in the traditional sense of diagnostic test evaluation. Instead, these intervention studies looked directly at patient outcomes, assuming that if the patient recovered without antibiotics then there was no serious bacterial illness. Although not being a new "gold standard", this circumvented the problem of the non-existent diagnostic "gold standard" to decide on the presence or absence of a clinically relevant bacterial infection based on traditional criteria.

## Conclusion

In conclusion, signs and symptoms routinely attributed to CAP are of limited value for the diagnosis of CAP. PCT and hsCRP can improve the diagnostic value of the clinical assessment. If confirmed, both parameters may, thus, be considered to replace leukocyte count in future guidelines of CAP. PCT has the highest diagnostic accuracy in differentiating radiographically confirmed CAP from other differential diagnoses, to predict bacteremia and to assess the severity of CAP.

## Abbreviations

CAP: Community-acquired pneumonia

PCT: Procalcitonin

AECOPD: Acute exacerbation of chronic obstructive pulmonary disease

PSI: Pneumonia severity index

QoL: Quality of life

BAL: Bronchoalveolar lavage

hsCRP: highly sensitive C-reactive protein

PCR: Polymerase chain reaction

ROC: Receiver operating characteristic curve

AUC: Areas under the receiver operating characteristic curve

## Competing interests

B Müller has served as consultant and received payments from Brahms (the manufacturer of procalcitonin assays) to attend meetings related to the trial and for travel expenses, speaking engagements, and research. S Harbarth has received speaker honoraria and research funding by Brahms. All other co-authors declare no conflict of interest.

## Authors' contributions

MCC had the idea of the study, drafted the protocol, collected and analyzed data, and wrote the report. BM had a substantial part in interpretation of the data, data collection and writing of the report. SH did the statistical analyses and had a substantial part in interpretation of the data and writing of the manuscript. DS, RB, JL, CM, and MT had substantial contributions in planning of the study, data collection, interpretation of data and/or writing of the manuscript. CN oversaw laboratory analysis. All authors read and approved the final manuscript.

## Pre-publication history

The pre-publication history for this paper can be accessed here:


